# Performance of Road-Traffic-Based Exposure Proxies Against Personal PM_2.5_ Measurements in Three Sub-Saharan African Countries

**DOI:** 10.64898/2026.03.13.26348337

**Published:** 2026-03-17

**Authors:** Handsome Bongani Nyoni, Terence Darlington Mushore, Laura Munthali, Sibusisiwe Audrey Makhanya, Laurine Chikoko, Stanley Luchters, Matthew F Chersich, Fortunate Machingura, Liberty Makacha, Benjamin Barratt, Hiten D Mistry, Marie Laure Volvert, Peter von Dadelszen, Anna Roca, Umberto D’alessandro, Marleen Temmeran, Esperança Sevene, Tamara Govindasamy, Prestige Tatenda Makanga, The PRECISE Network, The HEAT Centre

**Affiliations:** 1 Place Alert Labs, Surveying and Geomatics Department, Faculty of the Built Environment, Art and Design, Midlands State University, Gweru, Zimbabwe,; 2 Department of Architecture, Planning and Geomatics, University of Cape Town, Cape Town 7700, South Africa,; 3 Climate Environment and Health Department, Center for Sexual Health and HIV AIDS Research (CeSHHAR), Harare, Zimbabwe,; 4 IBM Research Africa, South Africa,; 5 Department of International Public Health, Liverpool School of Tropical Medicine (LSTM), Liverpool, UK,; 6 Department of Women and Children’s Health, School of Life Course and Population Sciences, Faculty of Life Sciences & Medicine, King’s College London, United Kingdom; 7 Environmental Research Group, MRC Centre for Environment and Health, Michael Uren Biomedical Engineering Hub, White City Campus, Imperial College London, United Kingdom,; 8 Department of Obstetrics and Gynaecology, University of British Columbia, Vancouver, British Columbia, Canada,; 9 Department of Public Health and Primary Care, Ghent University, Belgium; 10 Department of Space Science and Applied Physics, Faculty of Sciences, University of Zimbabwe,; 11 Malawi Liverpool Wellcome Programme, Public Health Group, Blantyre, Malawi,; 12 Medical Research Council Unit The Gambia at the London School of Hygiene and Tropical Medicine, Fajara, The Gambia; 13 Centre of Excellence Women and Child Health, Aga Khan University, Nairobi, Kenya; 14 Eduardo Mondlane University, Faculty of Medicine and Centro de Investigação em Saúde da Manhiça (CISM), Mozambique; 15 Department of Public Health and Epidemiology, College of Life Sciences, University of Leicester,; 16 Trinity College, Dublin, Ireland; 17 Wits Planetary Health Research, Wits Health Consortium, South Africa

## Abstract

**Introduction:**

Particulate Matter (PM_2.5_) exposure contributes to the global disease burden, yet its monitoring remains sparse and uneven and is limited in many limited ground monitoring network settings. Road-traffic proxy indicators can provide indirect estimates of PM_2.5_ where measurements are limited but require context-specific validation. We evaluated three PM_2.5_ road-traffic related proxies:(I) population-Weighted Road Network Density (WRND), (ii) Euclidean (straight line) distance from highways (EH), and (iii) Euclidean distance from main roads (EM).

**Methods:**

We validated proxies using high-resolution outdoor filtered PM_2.5_ personal exposure measurements collected over 1 year from 343 postpartum participants in The Gambia, Kenya, and Mozambique. Village-level spatial patterns for the PM_2.5_-proxy relationship were mapped using 5 km hexagonal aggregated tessellations. Proxy-PM_2.5_ associations were assessed using Spearman correlation, and predictive utility was tested using country-specific and global Random Forest (RF) models (3-fold cross-validation), reporting R^2^, RMSE, and feature importance

**Results:**

Spatial mapping showed heterogeneous proxy–PM_2.5_ relationships across and within sites, with elevated PM_2.5_ occurring in both low- and high-proxy contests. WRND–PM_2.5_ correlations were weak overall and statistically significant only in Mozambique (r = 0.351; *p = 0.005*), with non-significant associations in Kenya (r = −0.041; *p = 0.673*) and The Gambia (r = −0.020; *p = 0.909*). EH–PM_2.5_ correlations were positive in The Gambia (r = 0.335; *p = 0.053*) and Mozambique (r = 0.292; *p = 0.020*) but negative and significant in Kenya (r = −0.224; *p = 0.018*). Single-variable RF models performed poorly across all countries (R^2^ < 0.45) and the Global model (R^2^=0.42). Combining proxies improved performance in Kenya (R^2^=0.52; RMSE=31.7μg/m^3^) and Mozambique (R^2^=0.60; RMSE=8.9 μg/m^3^), Global R^2^=0.46; RMSE=29.1 μg/m^3^), although in The Gambia, the combined model (R^2^=0.53; RMSE=37.6 μg/m^3^) did not exceed the best single-proxy model.

**Conclusion:**

Road-network proxies provide context-dependent signals of personal PM_2.5_ exposure, and predictive performance is strengthened when proxies are combined in a hybrid model.

## 1 Introduction

Exposure to ambient and household air pollution, including fine Particulate Matter with an aerodynamic diameter ≤2.5 micrometers (PM_2.5_), remains a significant global public health concern in the 21st century. PM_2.5_ exposure contributes to an estimated 6.7 million premature deaths annually (1,2). These deaths are driven by cardiopulmonary pathways, including asthma, hypertensive disorders of pregnancy such as pre-eclampsia, cardiovascular disease, and a range of acute and chronic respiratory outcomes (3–5). The substantial health burden is partly attributable to PM_2.5_ size and chemical composition, which facilitate deep penetration into the respiratory tract, translocation across biological barriers, and systemic inflammation that affects multiple organ systems (3,4,6). Emerging evidence also links prenatal PM_2.5_ exposure to preterm birth, low birth weight, and impaired neurodevelopment among children (7,8). While a growing body of recent works suggests associations with poor mental well-being and general health (9–11). These impacts are amplified in sub-Saharan Africa (SSA) by rapid urbanization, increased motorization, unpaved-road dust, climate change, primary production, and landscape fire smoke, which collectively intensify both primary emissions and secondary particle formation (12,13). Effective management and mitigation of PM_2.5_ exposure depend on reliable air quality monitoring stations capable of capturing spatiotemporal variability (14). However, monitoring coverage is highly unequal in global villages.

High-income countries (HIC) typically have dense ground-based monitoring stations that provide broader spatio-temporal coverage. Low- and middle-income countries (LIMCs), particularly in Sub-Saharan Africa (SSA), remain severely under-monitored. According to the WHO ambient air pollution database of 2024, SSA accounts for less than 1% of global monitoring stations (203/40,222) (15). This reflects constraints, including limited policy enforcement, high setting up and operational costs, and shortages of the expertise required for sustained operation of a ground monitoring network (16). This results in limited spatiotemporal coverage of air pollution data, limiting exposure characterization for health assessment, and weakening the design of effective interventions and mitigation strategies (17). This persistent data gap has accelerated interest in alternative air pollution estimation methods, including proxy indicators for air pollution (18).

Proxy indicators are indirect measures used to infer air pollution/quality, including PM_2.5_ concentration, in regions without direct air quality measurements (19). Such indicators leverage open geospatial datasets, such as road network density, visibility, meteorological variables, and land-use/land-cover patterns, to characterize air pollution and infer pollutant concentrations (20). Road network-related proxies are of particular interest as traffic and associated resuspension are important contributors to urban PM_2.5_, and because road data are widely accessible (17). Additionally, literature suggests that integrating traffic type, volume, and distance to major routes using a linear regression model strongly predicts PM_2.5_ concentrations (21). However, while these proxies are widely applied in HIC, their validity in SSA remains insufficiently evaluated. SSA exhibits heterogeneous sources of PM_2.5;_ which cannot be quantified and assessed based on existing sparse ground monitoring systems. Robust validation of road and traffic related PM_2.5_ proxy indicators is therefore necessary to determine whether they can reliably support pollution exposure characterization in SSA.

In this study, we evaluated the validity of three road-network-based proxies for PM_2.5_ exposure: (i) population-weighted Road Network Density (WRND), (ii) Euclidean distance to Highway (EH), and (iii) Euclidean distance from Main roads (EM). The main aim of this study was to evaluate how these proxies performed against measured outdoor-filtered PM_2.5_ personal exposure data from The Gambia, Mozambique, and Kenya, collected as part of the PREgnancy Care Integrating Translational Science, Everywhere PRECISE- DYAD project (22,23).

## 2 Methods

### 2.1 Overall framework

**Figure 1.**
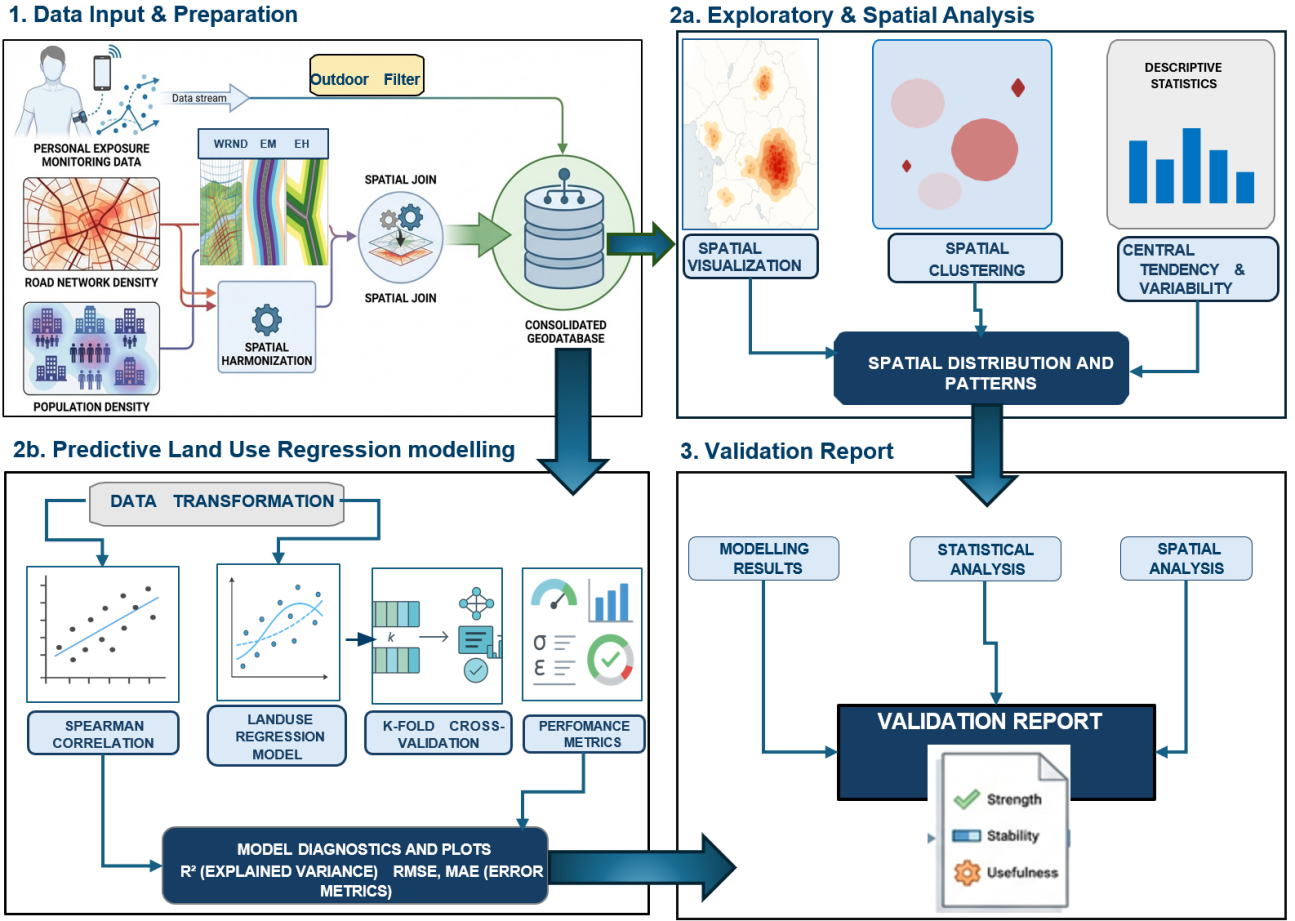
Flow chart of the validation framework developed in the study to validate the proxy indicators in Kenya, The Gambia, and Mozambique.

We developed a framework to validate road-traffic proxy indicators using high-resolution personal PM_2.5_ exposure data (Fig. 1). The framework integrates personal PM_2.5_ measurements from the PREgnancy Care Integrating Translational Science Everywhere (PRECISE-DYAD) project, road network data from OpenStreetMap, and gridded population data from WorldPop. All analyses in the study followed the Characterizing Effects of Air Quality In Maternal, Newborn and Child Health (CHEAQI-MNCH) protocol(24), and the use of PRECISE DYAD and PRECISE-HOME data complied with project ethical clearance and guidance.

While the PRECISE-DYAD personal monitoring protocol captures integrated exposure across indoor and outdoor micro-environments, this analysis restricted observations to outdoor movement periods to better isolate the traffic-related component relevant to proxy evaluation. By evaluating these proxies against outdoor PM_2.5_ exposure measurements across multiple SSA settings, we provide a conceptual framework for PM_2.5_ estimation in regions with sparse conventional air quality networks.

### 2.2 Study Sites

**Figure 2:**
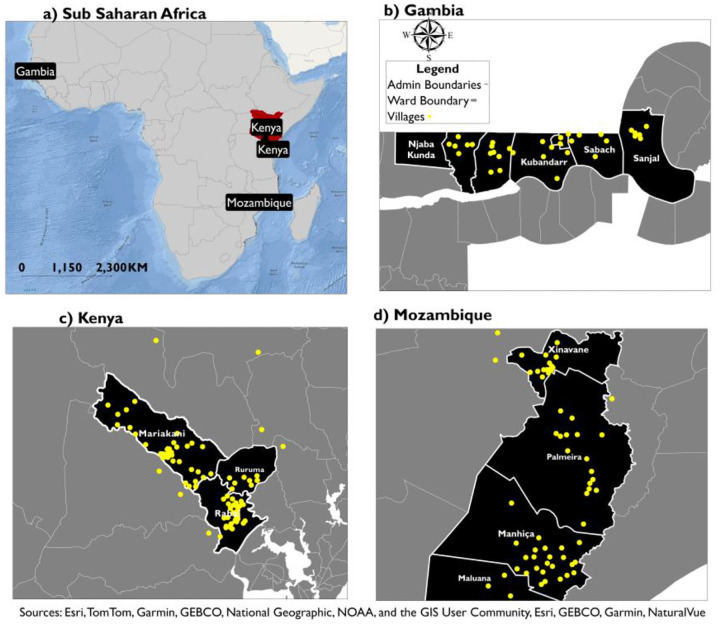
PRECISE study villages located in Kenya, Mozambique, and The Gambia, representing East, West, and Southern Africa regions. Data used to validate proxy indicators was collected in these villages.

The study was conducted in selected villages in three African countries: southern Mozambique, coastal Kenya, and the North Bank region of The Gambia (25,26) (Fig 2). These sites represent contrasting SSA geographies, road connectivity, settlement form, and land use contexts that influence spatial patterns of PM_2.5_ exposure (27,28). In Mozambique, the study villages are located within Manhica District and Xinavane locality, both in Maputo province, an area characterized by intensive large-scale sugarcane agricultural activity (29). In Kenya, the study sites fall within the coastal corridor around Mariakani, and the rural Rabai Sub-County located along the northern corridor, where heavy traffic volumes are a major potential source of PM_2.5_ (30). The Gambia, the study sites are situated in and around Farafenni and within surrounding rural districts, including Illiasa and Sabach Sanjal. These districts are characterized by dense population and a high level of informal transport (31). In general, ground air quality monitoring is limited in these countries and concentrated in a small number of locations, primarily in capital cities (Mozambique: 1 in Boane, Kenya: 1 in Nairobi and 1 in Mombasa, The Gambia: 1 in Banjul).

### 2.3 Data: Personal exposure and proxies

This study used a secondary high-resolution personal PM_2.5_ exposure dataset from the PRECISE-DYAD project comprising 1,048,576 one-minute observations collected over 12 months (2022–2023). In total, 343 women were recruited in PRECISE-DYAD to carry a personal exposure monitor bag that recorded PM_2.5_ concentrations (μg/m^3^), alongside relative humidity, and air temperature, capturing both indoor and outdoor microenvironments. However, this study focused on outdoor exposure as it better reflects road traffic-related pollution.

To identify outdoor mobility periods and reduce contamination from stationary and indoor time, we applied a spatial displacement threshold of 100 m/min (1.55 m/s). This threshold exceeds the commonly used static-cluster threshold (~0.83 m/s) used in other studies (32,33), thereby reducing misclassification of non-outdoor periods as outdoor. The selected value was consistent with typical adult walking speed (1.0–1.8 m/s) (34,35), supporting the use of 1.5 m/s to identify sustained outdoor movement.

To improve the robustness of this classification, a spatial validation step was implemented using footprint polygons from OpenStreetMap. Building geometries were retrieved through the Overpass API and used to perform point-in-polygon analysis to determine whether monitoring points occurred inside or outside building footprints. Speed-based classifications were compared with building-based labels using confusion matrix analysis, and discrepancies were corrected using the building-derived classification as ground truth to minimize misclassification of indoor and outdoor environments. After applying these filtering and validation procedures, the recorded observations were retained for subsequent exposure analysis.

We derived a population-weighted road network density (WRND) indicator by integrating road density with population distribution. The inputs included OpenStreetMap road network data (May 2024), village boundary polygons, village centroid point locations, and WorldPop (2018) gridded population data at 100m spatial resolution. We projected the road-network dataset to WGS84 UTM coordinate system to support spatial distance and area calculations. We used ArcMap 10.8 to compute WRND at the neighborhood level by first clipping the road network to community boundaries and generating a regular fishnet grid aligned to the 100m population raster. The total road length within each grid cell was summarized using the cell ID as the aggregation unit, and road network density (RND) was calculated per unit area (km/km^2^). Population counts from WorldPop were extracted and assigned to the corresponding grid cell, producing a weighted Road Network Density score. The wRND score was computed using Eq. 1.


[Eq. 1] WRND_village=Σ(RND_cell×Population_cell)/Σ(Population_cell)

We also derived distance proxies in ArcMap 10.8 by calculating Euclidean (36) (straight-line) distances from village centroids to the nearest main roads (EM) and highways (EH) based on the OpenStreetMap road network. The road network was reprojected to WGS84 UTM Zones to ensure accurate metric distance estimation, and roads were classified by hierarchy to isolate main roads and highways for separate analysis. The Near tool was used to calculate the shortest straight-line distance from each village centroid to the closest road segment, identifying the nearest location along the road geometry.

### 2.4 Data Analysis: Spatial Association and Predictive Modelling of Personal PM_2.5_ Exposure Using Proxy Indicators

We mapped the spatial distribution of the proxy-PM_2.5_ relationship using proportional symbols in ArcMap 10.8. For each study region, a 5 km hexagonal tessellation grid (37) was generated, and village locations were spatially joined to the tessellation to aggregate village-level attributes within each hexagon. Within each hexagon, PM_2.5_ and proxy indicators (WRND, EM, and EH) were summarized using the mean, producing an aggregated surface of village clusters rather than individual village points. Proxies were then visualized as proportional symbol sizes, while PM_2.5_ was represented using a sequential color gradient (μg/m^3^), enabling comparison of PM_2.5_ intensity across proxy ranges within and between study regions. Proxy-PM_2.5_ associations were evaluated using Spearman’s rank correlation (38).

We modeled PM_2.5_ as a function of road-traffic proxies WRND, EH, and EM, fitting them individually and jointly within a Land Use Regression (LUR) model Framework. LUR is widely used to estimate spatial variations in pollutant concentrations using proxy geospatial predictors (39,40). LUR was implemented using a random forest (RF) regressor and an ensemble method that combines multiple decision-tree models on bootstrapped samples and averages predictions to reduce variance and overfitting (41). At each node split within a tree, RF considers a random subset of predictors (*mtry*), which decorrelates between trees and improves robustness.

**Figure 3:**
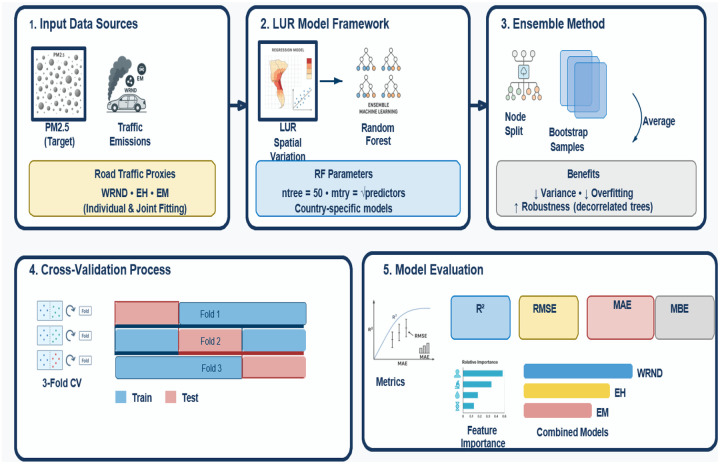
Diagrammatic representation of the random forest framework used to estimate PM_2.5_ across the study sites. For each country, four models were fitted (WRND, EH, EM, and Combined), each with 50 trees. Model outputs for PM_2.5_ were averaged across trees within each model and evaluated separately.

Country-specific RF models were developed for the study countries (Fig. 3). We set the number of trees (ntree) to 50 for computational efficiency and *mtry* to the square root of the number of predictors. The model performance was evaluated using 3 cross-validation folds and summarized by the coefficient of determination (R^2^), root mean squared error (RMSE), mean absolute error (MAE), and mean bias error (13,42,43). Feature importance was computed for the combined models.

## 3 Results

### 3.1 Proxies and Personal PM_2.5_ exposure – Summary statistics and spatial distribution

Kenya recorded the highest mean WRND (74.8), alongside PM_2.5_ mean = 31.3 μg/m^3^ (max = 173.1 μg/m^3^), EM mean = 0.7 km, and EH mean = 1.3 km (max = 42.5 km) - (Table 1). Mozambique showed the highest mean EH (4.0 km), with PM_2.5_ mean = 19.6 μg/m^3^ (max = 60.7 μg/m^3^), WRND mean = 16.1, and EM mean = 1.1 km. The Gambia displayed intermediate proxy levels (WRND mean = 26.1; EM mean = 1.5 km; EH mean = 1.8 km) but the highest PM_2.5_ burden overall (PM_2.5_ mean = 49.4 μg/m^3^; max = 220.7 μg/m^3^)

**Figure 4:**
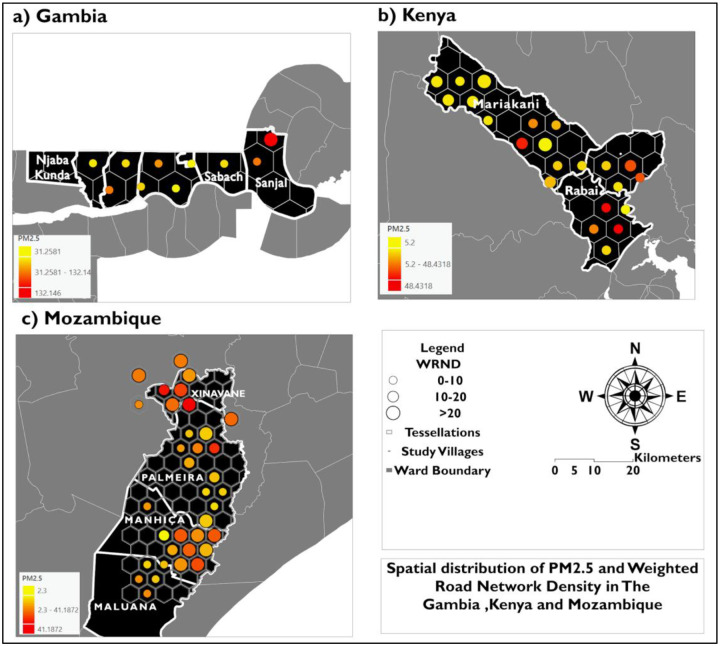
Spatial distribution of aggregated PM_2.5_ and weighted road network density (WRND) across hexagonal tessellations in (a) The Gambia, (b) Kenya, and (c) Mozambique. Hexagons represent aggregation units of the study villages. The yellow-to-orange color gradient represents PM2.5 (μg/m^3^), and proportional symbols represent WRND classes (0–10, 10–20, and >20) within each hexagon.

**Figure 5:**
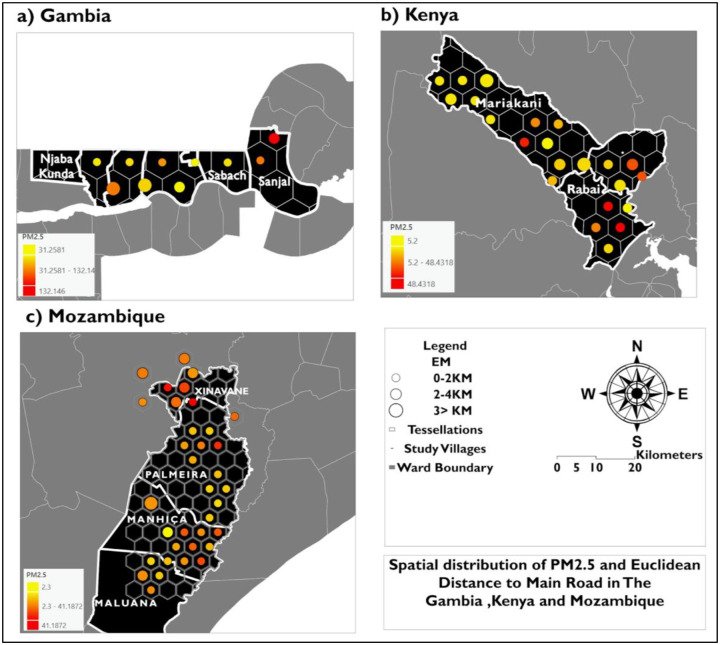
Spatial distribution of aggregated PM_2.5_ and Euclidean distance to main roads (EM) across hexagonal tessellations in (a) The Gambia, (b) Kenya, and (c) Mozambique. Hexagons represent aggregation units of the study villages. The yellow-to-orange color gradient represents mean PM_2.5_ (μg/m^3^), and proportional symbols represent mean EM classes (0–2 km, 2–4 km, and >3 km) within each hexagon.

The bivariate spatial distribution of PM_2.5_ and WRND across the village study villages aggregated into tessellations. is shown in Figure 4 In The Gambia (Fig. 4a), villages along Njaba Kunda/No Kunda and Farafenni have average to higher PM2.5 concentrations, ranging from low to moderate (0–20). In Kenya (Fig. 4b), villages in Mariakani and Rabai show mixed spatial patterns in which elevated PM_2.5_ occurs across multiple WRND classes. In Mozambique (Fig. 4c), Xinavane exhibits co-located higher values of PM_2.5_ and WRND; these clusters extend through some villages in Palmeria but occur across both lower and higher WRND classes.

the spatial distribution of the PM_2.5_-EM relationship in the study villages. is shown in figure 5 In The Gambia (Fig. 5a), villages in Sabach and Farafenni show higher PM_2.5_ concentrations, including low EM classes, while villages more than 20 km from Sanjal show elevated PM_2.5_ at greater distances. In Kenya (Fig. 5b), Mariakani and Rabai exhibit mixed patterns in which higher PM_2.5_ occurs in both low EM (0–2 km) and intermediate EM (2–4 km) hexagons. The results highlight that in Mozambique (Fig. 5c), villages in Xinavane exhibit higher PM2.5 concentrations across multiple EM classes, including some hexagons with larger EM (>3 km), contradicting a distance-decay effect in PM2.5 with increasing EM. Overall, across the study villages, the PM_2.5_–EM relationship is heterogeneous across and within sites, consistent with context-dependent proxy performance.

**Figure 6:**
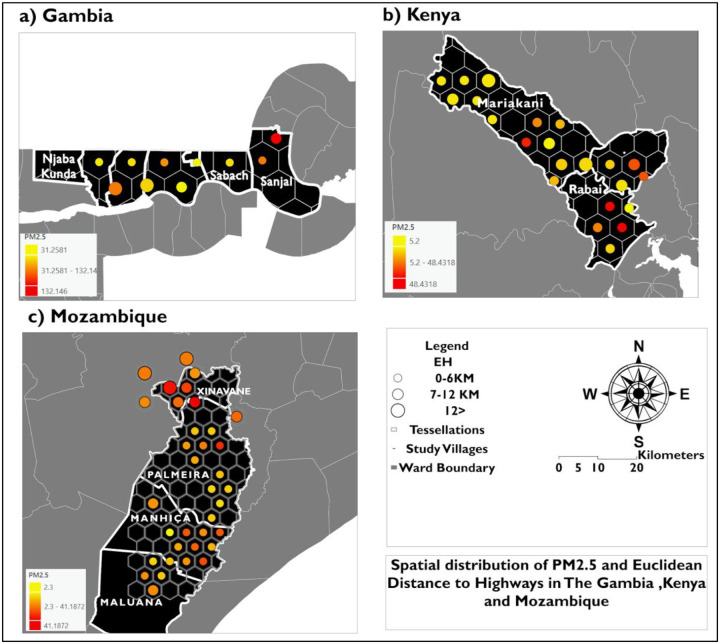
Spatial distribution of aggregated PM_2.5_ and Euclidean distance to highways (EH) across hexagonal tessellations in (a) The Gambia, (b) Kenya, and (c) Mozambique. Hexagons represent the aggregation units. The yellow to orange color gradient represents mean PM_2.5_ (μg/m^3^), and proportional symbols represent mean EH (km) within each hexagon.

The spatial distribution of the PM_2.5_–EH relationship in the study villages, is shown in figure 6 as hexagon tessellations. In The Gambia corridor villages in Njaba Kunda and Sabach show moderately elevated PM_2.5_ at low EH, aligning more closely with the proximity-to-highways assumption. In Kenya (Fig. 6b), Mariakani shows a proximity effect, with aggregated low-EH values corresponding to higher PM_2.5_ concentrations. In contrast, village Rabai shows higher PM_2.5_ at higher EH.

In Mozambique (Fig. 6c), Xinavane shows a direct positive relationship between higher PM_2.5_ and greater highway distances, contradicting the expected decline in PM_2.5_ with increasing EH.

**Figure 7:**
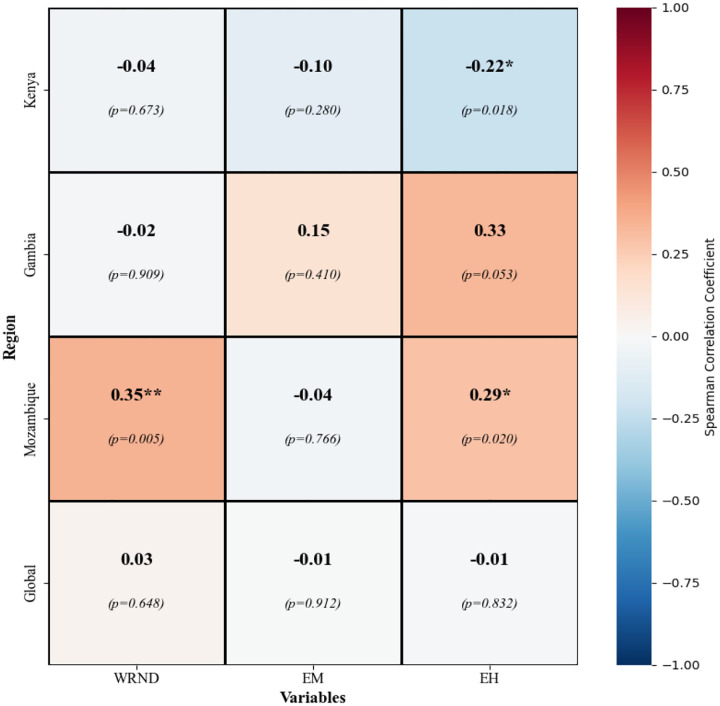
Spearman correlations between proxy indicators (WRND, EH EM) and pollutants (PM_2.5_) in Mozambique, Kenya, and the Gambia. The shades of blue indicate negative correlations, while those of red indicate positive correlations. Significance is denoted by asterisks: * p < 0.05, ** p < 0.01, *** p < 0.001.

Proxy-PM_2.5_ associations in Figure 7 indicate limited and context-specific statistical relationships. WRND was positively and significantly associated with PM_2.5_ in Mozambique (r = 0.351; *p = 0.005*), but associations were weak and non-significant in Kenya (r = −0.041; *p = 0.673*) and The Gambia (r = −0.020; *p = 0.909*); the pooled (“global”) association was near zero (r = 0.032; *p = 0.648*). EM showed weak, non-significant associations across all settings (Kenya r = −0.103; *p = 0.280*; The Gambia r = 0.146; *p = 0.410*; Mozambique r = −0.038; *p = 0.766*), with an overall weak negative Global association (r = −0.008; *p = 0.912*). EH exhibited contrasting behavior, with a significant negative association in Kenya (r = −0.224; *p = 0.018*) and a positive association in Mozambique (r = 0.292; *p = 0.020);* whilst association in The Gambia had positive but marginal (r = 0.335; *p = 0.053*).

### 3.2 Regression analysis of Personal PM_2.5_ exposure and proxy indicators

**Figure 8:**
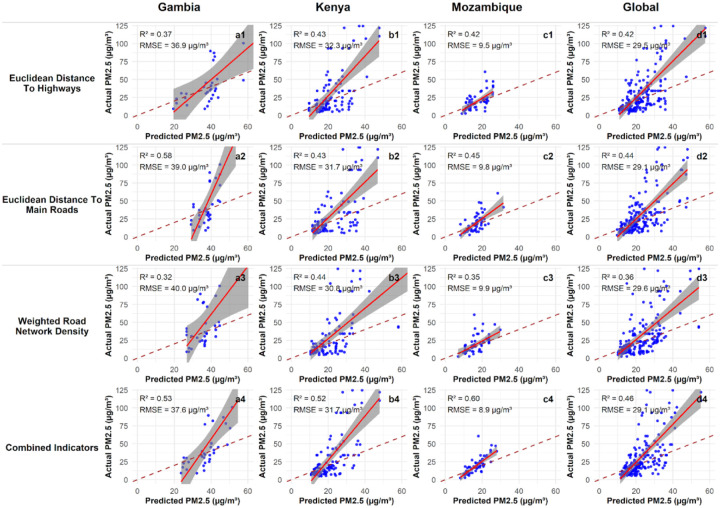
Observed versus predicted PM_2.5_ from Random Forest models using single and combined proxy indicators across study countries and the whole dataset. The panels show the PM_2.5_ model performance for each country, annotated with R^2^ and RMSE.

The observed versus predicted values for the RF models, using single and combined models across the study sites is shown in figure 8. The performance metrics of the models are in Table S2 and Figure S1.

EM only models’ regression ranged from The Gambia (R^2^=0.37; RMSE = 36.97 μg/m^3^), Kenya (R^2^ = 0.43; RMSE = 32.3 μg/m^3^), and Mozambique (R^2^ = 0.42; RMSE= 9.5μg/m^3^) and Global performing moderately as well (R^2^=0.42, RMSE= 32.3μg/m^3^). EH-PM_2.5_ models were the strongest single proxy in performance (R^2^= 0.58; RMSE = 39.0 μg/m^3^) and remained comparable in Kenya (R^2^ = 0.43; RMSE = 31.7 μg/m^3^) and Mozambique (R^2^ = 0.45; RMSE = 9.8 μg/m^3^), with Global R^2^ = 0.44 (RMSE = 29.1 μg/m^3^).

WRND-only models showed weaker performance in The Gambia (R^2^ = 0.32; RMSE = 40.0 μg/m^3^) but were like EM/EH in Kenya (R^2^= 0.44; RMSE = 30.8 μg/m^3^) and moderate in Mozambique (R^2^= 0.35; RMSE = 9.9 μg/m^3^), with Global R^2^ = 0.36 (RMSE = 29.6 μg/m^3^). Combining indicators improved performance in Kenya (R^2^ = 0.52; RMSE = 31.7 μg/m^3^) and Mozambique (R^2^ = 0.60; RMSE = 8.9 μg/m^3^) and increased pooled performance (Global R^2^= 0.46; RMSE = 29.1 μg/m^3^), although in The Gambia the combined model (R^2^ = 0.53; RMSE = 37.6 μg/m^3^) did not exceed the EM-only model.

**Figure 9:**
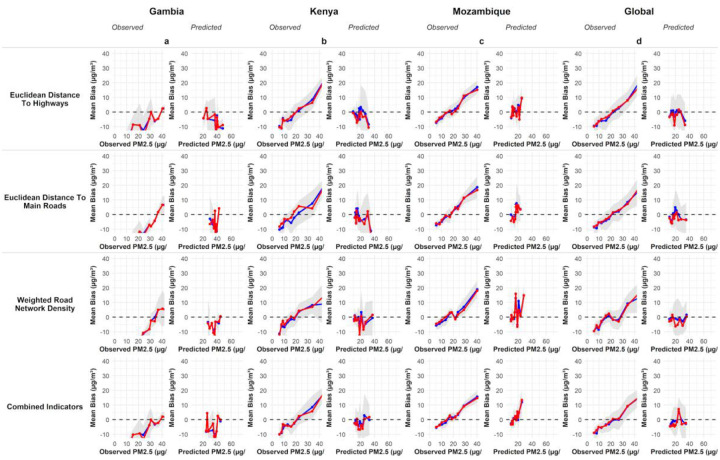
Mean bias error (MBE) plots for random forest PM_2.5_ predictions across study countries and the global dataset. The black line shows mean bias, the red line shows median bias, and the grey shading indicates the 95% confidence interval for mean bias. The plots show that combined proxy models exhibit the lowest overall bias, though bias increases to +10 μg/m^3^ at the highest concentrations.

Mean bias Error (MBE) plots for PM_2.5_ are shown in Fig.9. Combined proxy models showed the smallest and most stable biases across countries in Kenya; the combined biases ranged from mean ±2–5μg/m^3^ for low and mid-range(0–40μg/m^3^) concentration bins. Whilst in The Gambia and Mozambique, MBE exceeded 40 μg/m^3^ in the combined model at higher PM2.5 concentrations; the deviations were +20 μg/m^3^ at higher-range concentrations.

## 4 Discussion

In this study, we evaluated the validity of road traffic-related proxy indicators in estimating PM_2.5_ pollution exposure across three SSA countries (The Gambia, Kenya, and Mozambique), characterized by limited monitoring infrastructure. Beyond validating the study site-specific validation, our analysis provides a structured framework for evaluating the utility of proxy indicators in representing PM_2.5_ in areas with limited monitoring infrastructure.

### 4.2 Spatial distribution of proxy indicators and Personal PM_2.5_ exposure

In The Gambia, areas characterized by higher WRND and low EM exhibited higher PM_2.5_ concentrations, consistent with evidence linking dense road networks with elevated PM_2.5_ levels in urban and peri-urban environments (44). Similar spatial relationships between proximity to major roads and PM_2.5_ have been demonstrated in previous proximity-based analyses in Ireland (45).

Additionally, Traffic dispersion modelling in road-dense corridors yielded the same results, supporting the use of road proximity as a proxy of PM_2.5_ exposure (46). In contrast, Mozambique’s PM_2.5_ concentrations were less dependent on EM. They remained elevated even greater distances from main roads, highlighting that local sources were dominant and emanated from non-traffic processes. This finding mirrors findings from the same Xinavane region, where sugarcane burning is a major contributor to PM_2.5_ (47). In Kenya, higher PM_2.5_ levels from villages farther from main roads also suggest on-road traffic sources, as identified in other studies, including biomass burning and industrial activities (48).

### 4.3 Statistical Relationship of proxy indicators and Personal PM_2.5_ exposure

Overall, WRND showed moderate alignment with PM_2.5,_ in line with studies reporting positive road-PM_2.5_ associations in urban areas (49,50). However, weaker WRND-PM_2.5_ correlations in Kenya and The Gambia indicate that WRND alone is insufficient in some settings, suggesting stronger roles for land use, meteorology, and non-traffic emissions in shaping PM_2.5_ variability (51).

Notably, the positive correlation between PM_2.5_ and EH in The Gambia and Mozambique contradicts the typical distance-decay pattern observed in many LMICs (52). However, this reflects confounding non-traffic sources (biomass and agricultural burning) that elevate PM_2.5_ away from major highways. Overall, road proxies can inform PM_2.5_, but validity is context-dependent and improved by adding environmental covariates.

### 4.4 The regression analysis of Proxy indicators in predicting Personal PM_2.5_ exposure

Single proxy RF models showed limited predictive power for PM_2.5_ across all study sites, reflecting exposure variability unlikely to be captured by a single road-based variable. (53,54). Further, this finding aligns with studies in Italy, which also found that single proxy models had limited predictive capacity for PM_2.5._ (50). In particular, reliance on proximity-only measures (e.g., EM) demonstrated limited robustness, reflecting sensitivity to local spatial heterogeneity and shifting emission sources that complicate exposure modelling in urban and peri-urban environments (55).

In contrast, combining proxy indicators significantly improved PM_2.5_ prediction, highlighting the value of integrating spatial indicators, such as road network density and proximity to major roads, to represent exposure variability. This finding aligns with work from Cape Town, South Africa, and other LMIC regions, showing that multi-indicator models improve PM_2.5_ predictions relative to isolated proxies (56–58). Importantly, the pooled (“global”) analysis indicated moderate overall skill and further improvement when proxies were combined, suggesting that aggregating data across settings can stabilize estimation and capture shared proxy–PM_2.5_ structure that is not consistently recoverable within individual sites.

Nevertheless, predictive performance varied across countries, with weaker results in Kenya suggesting that road traffic proxies alone may not adequately capture PM_2.5_ spatio-temporal variability. This highlights the models’ limitations and the need for localized calibration and greater integration of environmental factors.

The feature of importance results further indicated that the dominant proxy contribution differs across countries. This reinforced the idea that proxy validity is context-specific and that modelling strategies should be tailored to local sources, mixtures, and spatial structures (59,60). Consistent with this are previous studies in Thailand that demonstrated that integrating satellite, environmental variables, and traffic data improves ground-level PM_2.5_ estimates, supporting a combined-data approach for exposure assessment (17).

### 4.5 Implications of Validation

These findings underscore the importance of validating proxy indicators of PM_2.5_ exposure. While some proxies demonstrated utility, inconsistent associations across countries indicate that single indicators are insufficient, and a multi-proxy approach is more robust. Prior evidence shows that incorporating meteorological variables, land-use patterns, and satellite-derived aerosol optical depth (AOD) data can improve estimates of PM_2.5_ exposure from diverse pollution sources (61,62).

Moreover, our study demonstrates the effectiveness of RF-based LUR models for estimating PM_2.5_ exposure in resource-constrained settings. However, between-country variability highlights the need for local calibration and expanding predictor sets where road proxies alone incompletely represent exposure dynamics.

## 5 Conclusion and Recommendations

This validation framework integrated personal exposure measurements with open geospatial data to assess the robustness of road-traffic proxies for PM_2.5_ in settings with limited monitoring. Overall, WRND proved to be the most consistent strong predictor of PM_2.5_. However, positive associations between EH and PM_2.5_ in The Gambia suggested significant contributions from non-traffic sources such as marketplaces. Underscoring the limits of pure road-based proxies. Spatial patterns also indicated elevated PM_2.5_ concentration both near and far from main and highway roads, highlighting the need for multi-source exposure models.

The LUR RF model results show that combining road proxies improved PM_2.5_ exposure estimates, but performance is likely to improve further through the integration of meteorological and remote-sensing covariates.

### Based on these findings, we recommend:

The development of hybrid models that combine LUR with satellite and meteorological datasets to improve PM_2.5_ exposure estimation in rapidly urbanizing regions of Sub-Saharan Africa. We also recommend expanding air quality personal and global monitoring sensor networks beyond urban centers to capture non-traffic PM_2.5_ pollution sources that would support calibration and validation. From this study, we have seen the need to integrate road traffic exposure models into urban planning to reduce vehicular emissions while promoting clean energy solutions. There is a need to test and scale this framework to extend PM_2.5_ exposure characterization across additional Sub-Saharan African countries.

## Supplementary Material

Supplement 1

Supplement 2

## Figures and Tables

**Table 1:** Summary statistics PM_*2*.5_ (μg/m^3^), WRND, EM(km), and EH(km) across study sites over the full analysis period.

Variable	Country	Mean	Std	Min	25%	Median	75%	Max
PM_2.5_	Gambia	49.4	42.0	9.0	24.5	35.5	66.5	220.7
	Kenya	31.3	35.7	4.8	9.6	16.3	34.1	173.1
	Mozambique	*19.6*	*11.8*	*2.3*	*10.4*	*18.2*	*24.1*	60.7
WRND	Gambia	26.1	25.1	5.9	17.0	20.7	25.7	152.5
	Kenya	74.8	46.8	*0.3*	41.5	75.0	98.2	198.0
	Mozambique	*16.1*	7.6	2.5	*8.8*	17.4	21.2	*36.5*
EM(km)	Gambia	1.5	1.6	0.2	0.4	0.7	2.1	6.7
	Kenya	0.7	*0.9*	*0.0*	*0.1*	0.5	0.9	7.0
	Mozambique	*1.1*	*1.0*	*0.0*	*0.5*	*0.7*	*1.5*	*5.4*
EH (km)	Gambia	1.8	1.6	0.3	0.5	1.7	2.5	*6.7*
	Kenya	1.3	4.2	*0.0*	*0.2*	*0.6*	1.2	42.5
	Mozambique	4.0	4.2	0.0	0.8	1.9	7.0	17.86

## References

[1] WHO. Household air pollution [Internet]. 2024 [cited 2025 Feb 6]. Available from: https://www.who.int/news-room/fact-sheets/detail/household-air-pollution-and-health

[2] Bachwenkizi J, Liu C, Meng X, Zhang L, Wang W, van Donkelaar A, Maternal exposure to fine particulate matter and preterm birth and low birth weight in Africa. Environ Int. 2022 Feb 1;160:107053. doi:10.1016/j.envint.2021.107053 34942408

[3] Adeoye M, Rahimzadeh S, Taylor S, Shrikhande S, Perel P, Shah A, The Impact of Air Pollution on Cardiovascular Health Outcomes in African Populations. JACC Adv. 2024 Dec;3(12):101371. doi:10.1016/j.jacadv.2024.101371 39817083 PMC11733974

[4] Jafta N, Shezi B, Buthelezi M, Muteti-Fana S, Naidoo RN. Household air pollution and respiratory health in Africa: persistent risk and unchanged health burdens. Curr Opin Pulm Med. 2025 Mar;31(2):89. doi:10.1097/MCP.0000000000001126 39410863 PMC11789611

[5] Vivien Wai Yun Lai, Lai VWY, Gayan Bowatte, Bowatte G, Luke D. Knibbs, Knibbs LD, Residential NO2 exposure is associated with urgent healthcare use in a thunderstorm asthma cohort. Asia Pac Allergy. 2018 Oct 10;8(4). doi:10.5415/apallergy.2018.8.e33. PMC6209594 30402400

[6] Biju Bárbara Pavani, B B, Vijayan N., N V. Estimation of Health Impact Due to AirPollution in Thiruvananthapuram City. Int J Innov Res Sci Eng Technol. 2014 Jan 1;3(7).

[7] Issah I, Duah MS, Arko-Mensah J, Bawua SA, Agyekum TP, Fobil JN. Exposure to metal mixtures and adverse pregnancy and birth outcomes: A systematic review. Sci Total Environ. 2024 Jan 15;908:168380. doi:10.1016/j.scitotenv.2023.168380 37963536

[8] Lavigne E, Yasseen AS, Stieb DM, Hystad P, van Donkelaar A, Martin RV, Ambient air pollution and adverse birth outcomes: Differences by maternal comorbidities. Environ Res. 2016 Jul 1;148:457–66. doi:10.1016/j.envres.2016.04.026 27136671

[9] Valencia Alejandro, Serre Marc L., Arunachalam Saravanan. A hyperlocal hybrid data fusion near-road PM2.5 and NO2 annual risk and environmental justice assessment across the United States. PLoS ONE. 2023 Jun 1;18(6):e0286406–e0286406. doi:10.1371/journal.pone.0286406. 37262039 PMC10234552

[10] Curto A, Donaire-Gonzalez D, Manaca MN, González R, Sacoor C, Rivas I, Predictors of personal exposure to black carbon among women in southern semi-rural Mozambique. Environ Int. 2019 Oct 1;131:104962. doi:10.1016/j.envint.2019.104962 31301586

[11] Adza Wisdom K., Hursthouse Andrew, Miller James, Boakye Daniel. Exploring the Combined Association between Road Traffic Noise and Air Quality Using QGIS. Int J Environ Res Public Health. 2022 Dec 19;19(24):17057–17057. doi:10.3390/ijerph192417057. 36554941 PMC9778687

[12] Greenwald R, Sarnat JA, Fuller CH. The impact of vegetative and solid roadway barriers on particulate matter concentration in urban settings. PLOS ONE. 2024 Jan 31;19(1):e0296885. doi:10.1371/journal.pone.0296885 38295020 PMC10830032

[13] Velizarova M., Dimitrova Reneta, Hristov Petar O., Burov Angel, Brezov D., Hristova Elena, Evaluation of Emission Factors for Particulate Matter and NO2 from Road Transport in Sofia, Bulgaria. Atmosphere. 2024. doi:10.3390/atmos15070773

[14] Claudia Falzone, Falzone Claudia, Romain AC, Romain Anne-Claude. Establishing an Air Quality Index Based on Proxy Data for Urban Planning Part 1: Methodological Developments and Preliminary Tests. Atmosphere. 2022 Sep 10;13(9):1470–1470. doi:10.3390/atmos13091470

[15] WHO Ambient Air Quality Database. WHO Ambient Air Quality Database (Update Jan 2024) [Internet]. 2024 [cited 2025 Feb 6]. Available from: https://www.who.int/publications/m/item/who-ambient-air-quality-database-(update-jan-2024)

[16] Khan AA, Kumar P, Gulia S, Khare M. A critical review of managing air pollution through airshed approach. Sustain Horiz. 2024 Mar 1;9:100090. doi:10.1016/j.horiz.2024.100090

[17] Baharane V, Shatalov AB. Assessment of the health impacts of air pollution exposure in East African countries. Environ Monit Assess. 2024 Apr 2;196(5):413. doi:10.1007/s10661-024-12588-0 38565772

[18] Wu Meng Hsing, Wu M, Ries Jean-Jacques, Ries JJ, Ries Jean-Jacques, Proietti Elena, Development of Late-Onset Preeclampsia in Association with Road Densities as a Proxy for Traffic-Related Air Pollution. Fetal Diagn Ther. 2016 Jan 1;39(1):21–7. doi:10.1159/000381802. 26088708

[19] Yun S, Zhong S, Alavi HS, Alahi A, Licina D. Proxy methods for detection of inhalation exposure in simulated office environments. J Expo Sci Environ Epidemiol. 2023 May;33(3):396–406. doi:10.1038/s41370-022-00495-w 36347935 PMC10234809

[20] Singh S, Kumar M, Verma BK, Kumar S. Optimizing Air Pollution Prediction With Random Forest Algorithm. Aerosol Sci Eng. 2025 Mar 3. doi:10.1007/s41810-025-00292-6

[21] Kumar P, Aishwarya, Srivastava PK, Pandey MK, Anand A, Biswas JK, Nitrogen dioxide as proxy indicator of air pollution from fossil fuel burning in New Delhi during lockdown phases of COVID-19 pandemic period: impact on weather as revealed by Sentinel-5 precursor (5p) spectrometer sensor. Environ Dev Sustain. 2024 Mar 1;26(3):6623–34. doi:10.1007/s10668-023-02977-9 PMC9907871 36785714

[22] The PRECISE-DYAD protocol: linking … | Wellcome Open Research [Internet]. [cited 2025 Dec 17]. Available from: https://wellcomeopenresearch.org/articles/7-281/v1

[23] Volvert ML, Wilson M, Owino RO, Koech A, Jah H, Blencowe H, Cohort Profile: PRECISE-DYAD: a prospective cohort study linking maternal and infant health trajectories in sub-Saharan Africa [Internet]. medRxiv; 2025 [cited 2026 Mar 12]. p. 2025.12.17.25342279. Available from: https://www.medrxiv.org/content/10.64898/2025.12.17.25342279v1 doi:10.64898/2025.12.17.25342279 PMC13384177 42468953

[24] Munthali L, Mushore TD, Nyoni HB, Makhanya SA, Aardenne van L, Makacha L, Characterizing effects of air quality in maternal, newborn and child health (CHEAQI–MNCH) in sub-Saharan Africa: a research protocol. J Glob Health Econ Policy. 2026 Jan 29;6. doi:10.7189/001c.155593

[25] von Dadelszen P, Flint-O’Kane M, Poston L, Craik R, Russell D, Tribe RM, The PRECISE (PREgnancy Care Integrating translational Science, Everywhere) Network’s first protocol: deep phenotyping in three sub-Saharan African countries. Reprod Health. 2020 Apr 30;17(1):51. doi:10.1186/s12978-020-0872-9 32354357 PMC7191688

[26] PREgnancy Care Integrating translational Science, Everywhere (PRECISE): a prospective cohort study of African pregnant and non-pregnant women to investigate placental disorders – cohort profile | BMJ Open [Internet]. [cited 2026 Mar 12]. Available from: https://bmjopen.bmj.com/content/15/5/e091831.info 10.1136/bmjopen-2024-091831 PMC12067852 40350193

[27] Tchanche Bertrand, Fomba Khanneh Wadinga, Mellouki Abdelwahid, Westervelt Daniel M., Giordano Michael R.. Scientists discuss the state of air quality research in Africa during the First International Conference on Air Quality in Africa – ICAQ’AFRICA2022. Clean Air J. 2022 Dec 22;32(2). doi:10.17159/caj/2022/32/2.15243

[28] Evangelopoulos D, Katsouyanni K, Keogh RH, Samoli E, Schwartz J, Barratt B, PM2.5 and NO2 exposure errors using proxy measures, including derived personal exposure from outdoor sources: A systematic review and meta-analysis. Environ Int. 2020 Apr 1;137:105500. doi:10.1016/j.envint.2020.105500 32018132

[29] Pinto J, Cossa N, Ferrari M, Coffey PS, Picolo M, Marrufo T, Integration and use of climate data by the national health system in Mozambique. J Clim Change Health. 2024 Dec 4;100368. doi:10.1016/j.joclim.2024.100368 PMC12851358 41646232

[30] Lazaro SAM, Baba VF. Air Pollution Resulting from Biomass Combustion in Mozambique: Origins, Consequences, and Measures for Mitigation. Environ Sci Proc. 2023;27(1):1. doi:10.3390/ecas2023-15117

[31] Egger EM, Salvucci V, Tarp F. Evolution of Multidimensional Poverty in Crisis-Ridden Mozambique. Soc Indic Res. 2023 Apr;166(3):485–519. doi:10.1007/s11205-022-02965-y 36999131 PMC10012298

[32] Full article: Understanding the vulnerability of multimodal public transportation networks [Internet]. [cited 2025 Dec 18]. Available from: https://www-tandfonline-com.ezproxy.uct.ac.za/doi/full/10.1080/10286608.2025.2601992?mi=iw3gv6

[33] SciSpace - Paper [Internet]. 2010 [cited 2025 Mar 1]. Mobility, accessibility and activity participation : a comparative assessment of methods to identify rural transport disadvantage. Available from: https://scispace.com/papers/mobility-accessibility-and-activity-participation-a-3yo7nyjofn

[34] Association between Walking Exercise and Physical Function: In Co [Internet]. [cited 2025 Dec 18]. Available from: https://www.longdom.org/open-access/association-between-walking-exercise-and-physical-function-in-communitydwelling-older-people-84712.html

[35] The metabolic equivalents of one-mile walking by older adults; implications for health promotion [Internet]. [cited 2025 Dec 18]. Available from: https://hpp.tbzmed.ac.ir/FullHtml/HPP_19676_20170822070501 10.15171/hpp.2017.38 PMC5647357 29085799

[36] Suwanda R, Syahputra Z, Zamzami EM. Analysis of Euclidean Distance and Manhattan Distance in the K-Means Algorithm for Variations Number of Centroid K. J Phys Conf Ser. 2020 Jun 1;1566(1):012058. doi:10.1088/1742-6596/1566/1/012058

[37] Gold C. Tessellations in GIS: Part I—putting it all together. Geo-Spat Inf Sci. 2016 Jan 2;19(1):9–25. doi:10.1080/10095020.2016.1146440

[38] Beckerman B, Jerrett M, Brook J, Verma D, Arain M, Finkelstein M. Correlation of nitrogen dioxide with other traffic pollutants near a major expressway. Atmos Environ. 2008 Jan 31;42:275–90. doi:10.1016/j.atmosenv.2007.09.042

[39] Ji Levy, Levy JI, Levy Jonathan I., JH Levy, Je Clougherty, Clougherty JE, Evaluating heterogeneity in indoor and outdoor air pollution using land-use regression and constrained factor analysis. Res Rep. 2010 Dec 1;(152):5–91. 21409949

[40] Mölter A, Lindley S. Developing land use regression models for environmental science research using the XLUR tool – More than a one-trick pony. Environ Model Softw. 2021 Sep 1;143:105108. doi:10.1016/j.envsoft.2021.105108

[41] Sharma V, Ghosh S, Dey S, Singh S. Modelling PM2.5 for Data-Scarce Zone of Northwestern India using Multi Linear Regression and Random Forest Approaches. Ann GIS. 2023 Jul 3;29(3):415–27. doi:10.1080/19475683.2023.2183523

[42] Hua Z, Sun W, Yang G, Du Q. A Full-Coverage Daily Average PM2.5 Retrieval Method with Two-Stage IVW Fused MODIS C6 AOD and Two-Stage GAM Model. Remote Sens. 2019 Jan;11(13):13. doi:10.3390/rs11131558

[43] Belitz K, Stackelberg PE. Evaluation of six methods for correcting bias in estimates from ensemble tree machine learning regression models. Environ Model Softw. 2021 May 1;139:105006. doi:10.1016/j.envsoft.2021.105006

[44] Yu C, Deng Y, Qin Z, Yang C, Yuan Q. Traffic volume and road network structure: Revealing transportation-related factors on PM2.5 concentrations. Transp Res Part Transp Environ. 2023 Nov 1;124:103935. doi:10.1016/j.trd.2023.103935

[45] Richmond-Bryant J, Chris Owen R, Graham S, Snyder M, McDow S, Oakes M, Estimation of on-road NO2 concentrations, NO2/NOX ratios, and related roadway gradients from near-road monitoring data. Air Qual Atmosphere Health. 2017 Jun;10(5):611–25. doi:10.1007/s11869-016-0455-7 PMC6145484 30245748

[46] Huang Shaodan, Huang S, Lawrence Joy, Lawrence J, Kang Choong-Min, Kang CM, Road proximity influences indoor exposures to ambient fine particle mass and components. Environ Pollut. 2018 Sep 17;243:978–87. doi:10.1016/j.envpol.2018.09.046. 30248605

[47] Wang F, Peng Y, Jiang C. Influence of Road Patterns on PM2.5 Concentrations and the Available Solutions: The Case of Beijing City, China. Sustainability. 2017 Feb;9(2):2. doi:10.3390/su9020217

[48] Omondi E, Iddi S, Chepkemoi S, Mugotitsa B, Cygu S, Okumu B, Understanding demographic events and migration patterns in two urban slums of Nairobi City in Kenya. Sci Rep. 2024 Nov 21;14(1):28852. doi:10.1038/s41598-024-79895-x 39572746 PMC11582661

[49] Jin L, Berman JD, Warren JL, Levy JI, Thurston G, Zhang Y, A land use regression model of nitrogen dioxide and fine particulate matter in a complex urban core in Lanzhou, China. Environ Res. 2019 Oct;177:108597. doi:10.1016/j.envres.2019.108597 31401375

[50] Shi T, Zhang Y, Yuan X, Li F, Yan S. Spatial Patterns and Determinants of PM2.5 Concentrations: A Land Use Regression Analysis in Shenyang Metropolitan Area, China. Sustainability. 2024 Jan;16(12):12. doi:10.3390/su16125119

[51] Ahmad M, Cheng W, Xu Z, Kalam A. Outlier Detection of Air Quality for Two Indian Urban Cities Using Functional Data Analysis. Open J Air Pollut. 2023 Aug 29;12(3):3. doi:10.4236/ojap.2023.123005

[52] Shams SR, Jahani A, Kalantary S, Moeinaddini M, Khorasani N. Artificial intelligence accuracy assessment in NO2 concentration forecasting of metropolises air. Sci Rep. 2021 Jan 19;11(1):1805. doi:10.1038/s41598-021-81455-6 33469146 PMC7815891

[53] Jin J, Liu S, Wang L, Wu S, Zhao W. Fractional Vegetation Cover and Spatiotemporal Variations of PM2.5 Concentrations in the Beijing-Tianjin-Hebei Region of China. Atmosphere. 2022 Nov;13(11):1850. doi:10.3390/atmos13111850

[54] Mathew A, Gokul PR, Raja Shekar P, Arunab KS, Ghassan Abdo H, Almohamad H, Air quality analysis and PM2.5 modelling using machine learning techniques: A study of Hyderabad city in India. Cogent Eng. 2023 Dec 31;10(1):2243743. doi:10.1080/23311916.2023.2243743

[55] Kamana AA, Radoine H, Nyasulu C. Urban challenges and strategies in African cities – A systematic literature review. City Environ Interact. 2024 Jan 1;21:100132. doi:10.1016/j.cacint.2023.100132

[56] Morapedi TD, Obagbuwa IC. Air pollution particulate matter (PM2.5) prediction in South African cities using machine learning techniques. Front Artif Intell. 2023 Oct 10;6. doi:10.3389/frai.2023.1230087 PMC10595005 37881653

[57] Ma X, Zou B, Deng J, Gao J, Longley I, Xiao S, A comprehensive review of the development of land use regression approaches for modeling spatiotemporal variations of ambient air pollution: A perspective from 2011 to 2023. Environ Int. 2024 Jan 1;183:108430. doi:10.1016/j.envint.2024.108430 38219544

[58] Ndletyana O., Madonsela B. S.. Spatial Distribution of PM10 and NO2 in Ambient Air Quality in Cape Town CBD, South Africa. Nat Environ Pollut Technol. 2023 Mar 2;22(1):1–13. doi:10.46488/nept.2023.v22i01.001

[59] Houdou A, Badisy IE, Khomsi K, Abdala SA, Abdulla F, Najmi H, Interpretable Machine Learning Approaches for Forecasting and Predicting Air Pollution: A Systematic Review. Aerosol Air Qual Res. 2024;24(1):230151. doi:10.4209/aaqr.230151 PMC13319251 42389239

[60] Padhy MP, Reddy MCS. AIR POLLUTION PREDICTION USING MACHINE LEARNING. Vol. 12. 2024;12(2).

[61] Tang P, Yang X, Sun X, Ye H. Correlation between NDVI and PM2.5 Concentrations in a Small-Scale Urban Area [Internet]. 2023 [cited 2024 Sep 16]. Available from: https://www.researchsquare.com/article/rs-3146587/v1 doi:10.21203/rs.3.rs-3146587/v1

[62] Philipp Franke A. Lange H. Elbern. Evaluation of European anthropogenic trace gas and aerosol emissions using 4D-var: First results of a full-year reanalysis for 2016. 2021.

