# Supplementary material for: Performance of Road-Traffic-Based Exposure Proxies Against Personal PM2.5 Measurements in Three Sub-Saharan African Countries
Source: medRxiv. 2026 Mar 17:2026.03.13.26348337. Preprint. [Version 1] doi: 10.64898/2026.03.13.26348337 (PMC13015645; doi:10.64898/2026.03.13.26348337)
Supplement: Supplement 2 [file media-2.pdf]

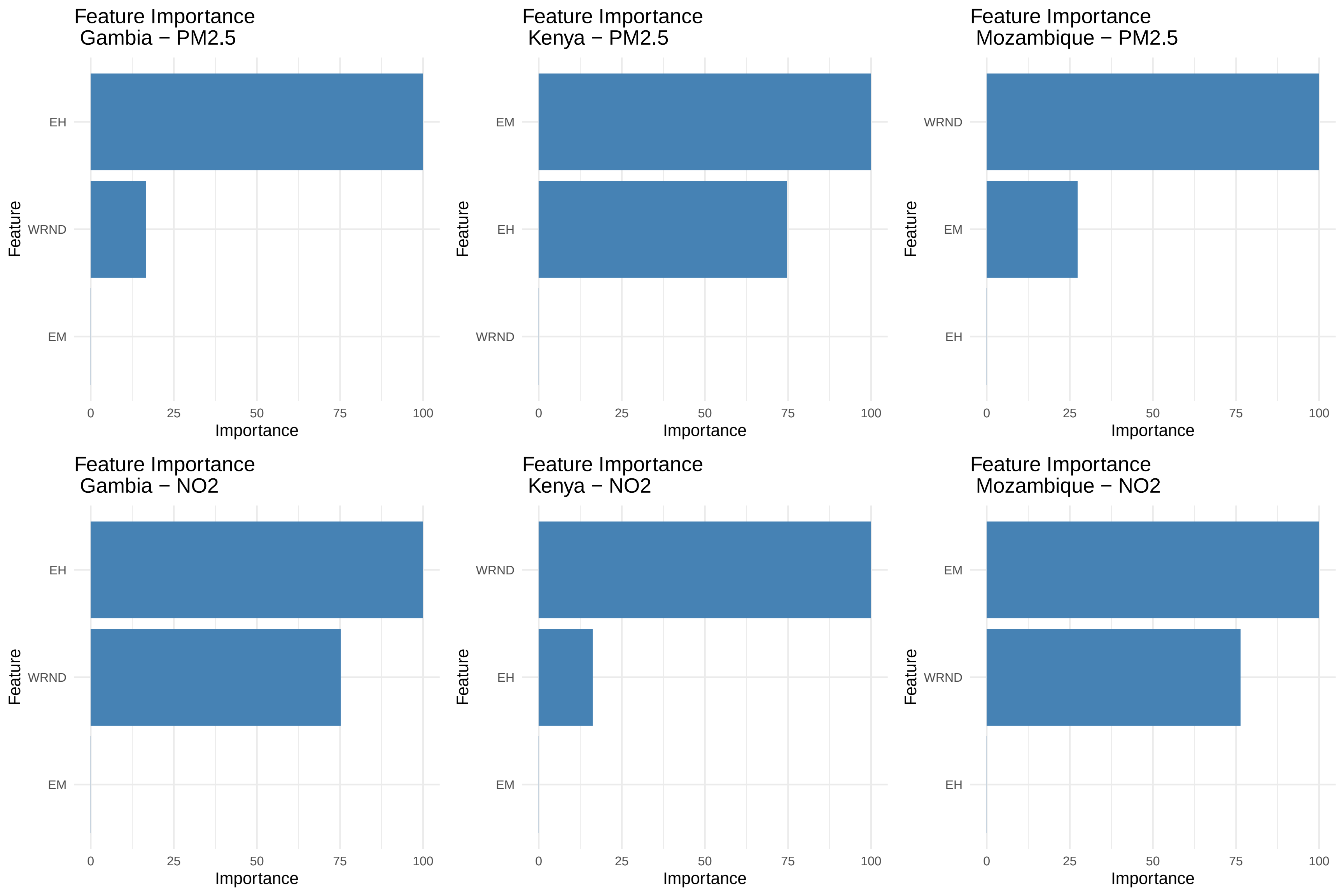
Feature Importance
Gambia − PM2.5
EH
WRND
EM
Feature
0
25
50
75
100
Importance
Feature Importance
Kenya − PM2.5
EM
EH
WRND
Feature Importance
Mozambique − PM2.5
WRND
EM
EH
Feature Importance
Gambia − NO2
EH
WRND
EM
Feature Importance
Kenya − NO2
WRND
EH
EM
Feature Importance
Mozambique − NO2
EM
WRND
EH
